# Associations Between Household Pesticide Exposure, Smoking and Hypertension

**DOI:** 10.3389/fpubh.2022.754643

**Published:** 2022-02-22

**Authors:** Haiyan Chen, Xiaohua Liang, Li Chen, Lei Zuo, Kuncai Chen, Yuehong Wei, Shouyi Chen, Guang Hao

**Affiliations:** ^1^Guangzhou Center for Disease Control and Prevention, Guangzhou, China; ^2^Ministry of Education Key Laboratory of Child Development and Disorders, National Clinical Research Center for Child Health and Disorders, Clinical Epidemiology and Biostatistics Department, Children's Hospital of Chongqing Medical University, Chongqing, China; ^3^Key Laboratory of Pediatrics in Chongqing, Chongqing, China; ^4^China International Science and Technology Cooperation Center of Child Development and Critical Disorders, Chongqing, China; ^5^Department of Medicine, Georgia Prevention Institute, Medical College of Georgia, Augusta University, Augusta, GA, United States; ^6^Department of Public Health and Preventive Medicine, School of Medicine, Jinan University, Guangzhou, China

**Keywords:** smoking, household pesticide exposure, hypertension, interaction, NHANES

## Abstract

This analysis aims to investigate the association between household pesticide exposure and hypertension risk, and to determine whether smoking plays a role in this association. We used data from the National Health and Nutrition Examination Survey (NHANES) for the years 1999–2014, including a total of 32,309 U.S. adult participants who were 20 years or older. Smoking status and pesticide exposure were self-reported. Blood pressure was measured by trained personnel using a mercury sphygmomanometer, according to a standardized protocol. We observed an increased risk of hypertension (OR [odds ratio] = 1.10, 95% confidence intervals [CI]: 1.01–1.18) in participants with exposure to household pesticides. Moreover, a significant interaction between smoking status and pesticide exposure on hypertension was observed (*P* = 0.022). Stratified analysis showed that household pesticide exposure was associated with a 29% higher risk of hypertension (OR = 1.29, 95% CI: 1.08–1.53) in smokers. However, for non-smokers, this association was not significant. Similar trends were found for systolic and diastolic blood pressures. In addition, we investigated the associations between pesticide metabolites in urine/serum and hypertension and found that several metabolites of dioxins, furans, and coplanar polychlorinated biphenyls were significantly associated with a higher risk of hypertension. This study suggests that household pesticide exposure is associated with an elevated risk of hypertension. We also report that smoking may accentuate the effect of pesticide exposure on hypertension.

## Introduction

Pesticides are widely used in households to control insects worldwide. Humans are exposed to these chemicals through direct dermal absorption, oral intake, or inhalation (1, 2). It is well-established that pesticide exposure has a critical influence on human health. Previous studies have reported that pesticide use is associated with various adverse health effects, including psychiatric disorders, fetal defects, and diabetes (3–6). Although emerging evidence suggests that pesticide exposures increase the risk of developing high blood pressure (BP) (7, 8), inconsistency between different studies still exists. For example, the Human Early-Life Exposome (HELIX) project did not observe an association between early-life exposure to organophosphate pesticides and BP in children (9). In contrast, in a cross-sectional study, Goncharov et al. reported that serum polychlorinated biphenyls (PCBs) concentration was strongly associated with elevated BP, but found little or no relationship between organochlorine (OC) pesticides and BP level (10). However, results from a population-based study in the Canary Islands (Spain) did not reveal any PCB congener as a hypertension risk factor. This study even reported that the cyclodiene pesticide aldrin was negatively associated with hypertension (11). A cross-sectional study involving 1,839 men and women aged 40–60 years also revealed that pesticide exposure was associated with reduced levels of BP levels in women (12).

Smoking is a well-known risk factor for hypertension. Previous studies reported an interaction effect between smoking and persistent organic pollutants (POPs), PCBs, or organochlorine pesticides on mortality (13, 14), indicating that smoking may modify the association between pesticides exposure and hypertension.

Thus, the present study aimed to evaluate whether household pesticide exposure is associated with elevated risks of high BP using data from the US National Health and Nutrition Examination Survey (NHANES). Furthermore, we investigated if smoking influenced the association between household pesticide exposure and hypertension risk.

## Methods

### Study Population

The NHANES is conducted by the National Center for Health Statistics (NCHS) and Centers for Disease Control and Prevention. It included a stratified multistage probability sample representative of the civilian non-institutionalized U.S. population. Detailed descriptions of the survey design and data collection procedures are available elsewhere (15). Since 1999, the demographic, socioeconomic, health-related data, etc., have been collected via an in-home interview and a visit to a mobile examination center. The research ethics boards of the NCHS approved all protocols and participants gave written informed consent to each examination.

We used data from eight cycles of the NHANES for the years 1999–2014. The following selection criteria were used: (1) aged 20 years or older; (2) complete data on variables of age, sex, race, body mass index (BMI), recreational physical activity (RPA), smoking status, marital status, education attainment, poverty income ratio, BP, and pesticide exposure. Finally, a total of 32,309 U.S. adult participants were included in the study. The data used in the analysis are publicly available at https://www.cdc.gov/nchs/nhanes/index.htm.

### Measures

BP was measured by trained personnel using a mercury sphygmomanometer according to a standardized protocol. Three consecutive readings were taken for each participant. For the present analysis, the average of three readings was used. Hypertension was defined as a mean systolic BP of ≥130 mmHg, a mean diastolic BP of ≥80 mmHg, or as “use of BP-lowering medication,” according to the AHA/ACC 2017 hypertension guideline (16). To facilitate comparison with previous studies, we also reported the results in which hypertension was defined as a mean systolic BP of ≥140 mmHg, a mean diastolic BP of ≥90 mmHg, or as “use of BP-lowering medication” according to the old guideline (17). Blood and urine samples are collected and processed in the mobile examination center by certified laboratory professionals, then stored in biorepositories.

Household pesticide exposure was defined as the responses to the question “In the past 7 days, were any chemical products used in {your/his/her} home to control fleas, roaches, ants, termites, or other insects?” In addition, pesticide metabolites were assessed in participants on randomly selected subsamples (15). We explored the associations of dioxins, furans, and coplanar PCBs in serum (NHANES 1999–2000, 2001–2002, and 2003–2004), pyrethroids (NHANES 1999–2000, 2001–2002, 2007–2008, 2009–2010, 2011–2012, and 2013–2014), phenolic compounds (NHANES 2003–2004, 2005–2006, 2007–2008, 2009–2010, and 2011–2012), organophosphorus (NHANES 1999–2000, 2001–2002, 2003–2004, 2005–2006, 2007–2008, and 2011–2012), carbamates and sulfonylurea herbicides (NHANES 2005–2006 and 2007–2008) in urine with hypertension. Dioxins, furans, and coplanar PCBs were measured by high-resolution gas chromatography/isotope-dilution high-resolution mass spectrometry (HRGS/ID-HRMS). Urinary concentrations of pyrethroid metabolites, including 3-PBA, were determined by high-performance liquid chromatography combined with electrospray chemical ionization and tandem mass spectrometry using validated laboratory methods (18). Phenolic compounds were measured by using gas chromatography (GC) or high-performance liquid chromatography (HPLC) coupled with different detection techniques. Carbamates and Organophosphorus were measured by using lyophilization and chemical derivatization followed by analysis using isotope-dilution gas chromatography-tandem mass spectrometry (GC–MS/MS). Sulfonylurea herbicides were analyzed by high-performance liquid chromatography-tandem mass spectrometry using turbo-ion spray atmospheric pressure ionization (15).

Participants who had smoked > 100 cigarettes in their entire life and currently smoked were defined as “current smokers,” while others were “current non-smokers.” The measurements and definitions of other covariates were published previously (19).

### Statistical Analyses

Appropriate sample weights were used to account for oversampling and non-response to provide nationally representative results, as recommended by NHANES Analytic Guidelines. Continuous variables were presented as mean and standard deviation (SD), whereas categorical variables were presented as cases (n) and percentages (%). Chi-squared tests and 2-tailed Student's *t*-tests were used to compare the characteristics of the normal and hypertension groups. Logistic regression was used to calculate the odds ratio (OR) and 95% confidence interval (CI) for the association of pesticide exposure and hypertension. Linear regression was used to calculate the β and 95% CI for the association of pesticide exposure and BP (7,997 hypertensive patients under antihypertensive medication were excluded). Model 1 was a univariate analysis. Model 2 was adjusted for age, sex, and race. Model 3 was further adjusted for BMI, RPA, marital status, current smoking status, education attainment, and poverty income ratio. The interaction between pesticide exposure and smoking status on hypertension was investigated by adding a pesticides × smoking term in the model. Log-transformations were applied for all the metabolites, and multivariate logistic regressions were used to test their assertions with hypertension. All data analyses were performed using Stata software version 12.1 (STATA Corp., TX, US). A two-sided *P* < 0.05 was considered statistically significant.

## Results

There were 32,309 participants eligible for our final analysis, of which 4,467 (13.8%) self-reported exposure to a household pesticide (13.0% in normal group and 14.4% in hypertension group.). The mean age of participants was 48.71 ± 18.0 years; 50.7% of the participants were female; 49.4% of the participants had hypertension. The characteristics of hypertension and normal groups were significantly different, except for poverty income ratio (Table 1). Selected metabolites levels were presented in (Supplementary Table 1).

**Table 1:** Characteristics of the participants.

**Variable**	**Normal**	**Hypertension**	***P* value**
	**(*n* = 16,364)**	**(*n* = 15,945)**	
Age (years)	40.1 ± 15.4	57.6 ± 15.9	<0.001
Sex (Females, %)	9,010 (55.1)	7,376 (46.3)	<0.001
**Race (%)**			
Non-Hispanic white	7,774 (47.5)	7,864 (49.3)	<0.001
Non-Hispanic black	2,666 (16.3)	3,821 (24.0)	
Mexican-American	3,334 (20.4)	2,382 (14.9)	
Other	2,590 (15.8)	1,878 (11.8)	
Body mass index (kg/m^2^)	27.5 ± 5.9	30.0 ± 6.8	
Current smoke (%)	4,232 (25.9)	3,344 (21.0)	<0.001
Marital status (%)	8,610 (52.6)	8,927 (56.0)	<0.001
**Education attainment**			
<9 years	1,455 (8.9)	2,153 (13.5)	<0.001
9–11 years	2,401 (14.7)	2,569 (16.1)	
12 years	3,522 (21.5)	3,975 (24.9)	
>12 years	8,986 (54.9)	7,248 (45.5)	
Vigorous/Moderate RPA (%)	9,590 (58.6)	7,694 (48.3)	<0.001
Poverty income ratio	2.6 ± 1.7	2.6 ± 1.6	0.667
Self-reported pesticide exposure (%)	2,177 (13.0)	2,290 (14.4)	0.006
Systolic blood pressure (mmHg)	111.9 ± 9.4	135.6 ± 18.7	<0.001
Diastolic blood pressure (mmHg)	65.9 ± 9.6	74.2 ± 15.3	<0.001

*RPA, recreational physical activity*.

*Continuous variables are presented as mean ± standard deviation, and categorical variables are presented as cases (percentage)*.

The prevalence of hypertension in the pesticide-exposed and pesticide-unexposed groups were 51.3 and 49.0%, respectively. After adjusting for age, sex, and race, it was observed that participants who were exposed to household pesticides had a higher risk (*OR* = 1.08, 95% CI: 1.00–1.17, *P* = 0.050) of hypertension than those unexposed. Further adjusting for BMI, current smoking status, RPA, marital status, education attainment, and poverty income ratio, the results remained similar (*OR* = 1.10, 95% CI: 1.01–1.18, *P* = 0.020) (Table 2). When the hypertension was defined by old guidelines (systolic BP of ≥140 mmHg or diastolic BP of ≥90 mmHg), a similar positive association was observed (*OR* = 1.06, 95% CI: 0.96–1.17, *P* = 0.235) but it did not reach statistical significance (Supplementary Table 2).

**Table 2:** Associations between household pesticide exposure and hypertension.

	**OR**	**95% CI**	***P* value**
**Model 1**			
Control	Reference		
Pesticide exposure	1.04	0.97–1.13	0.206
**Model 2**			
Control	Reference		
Pesticide exposure	1.08	1.00–1.17	0.050
**Model 3**			
Control	Reference		
Pesticide exposure	1.10	1.01–1.18	0.020

*OR, Odds ratio; CI, confidence intervals*.

*Model 1 unadjusted*.

*Model 2 adjusted for age, sex, and race*.

*Model 3 adjusted for age, sex, race, body mass index, current smoking status, recreational physical activity, marital status, education attainment, and poverty income ratio*.

A significant interaction between smoking and household pesticide exposure on the risk of hypertension was observed (*P* = 0.022). Stratified analysis by smoking status showed that, for current smokers, the participants with pesticide exposure had a 29% higher risk of hypertension than those without exposure (adjusted *OR* = 1.29, 95% CI: 1.08–1.53, *P* = 0.004). However, the association was not significant (adjusted OR = 1.02, 95% CI: 0.93–1.12, *P* = 0.682) for non-smokers (Table 3). Moreover, we further investigated the associations between pesticide metabolites in urine/serum and hypertension. The results showed positive associations between several metabolites of dioxins, furans, or coplanar PCBs in serum and hypertension (Table 4), and null or negative associations for other metabolites (Supplementary Table 3).

**Table 3:** Associations between household pesticide exposure and hypertension by smoking status.

	**OR**	**95% CI**	***P* value**
**Smokers**			
Pesticide-unexposed	Reference		
Pesticide-exposed	1.29	1.08–1.53	0.004
**Non-smokers**			
Pesticide-unexposed	Reference		
Pesticide-exposed	1.02	0.93–1.12	0.682

*CI, confidence intervals; RPA, recreational physical activity*.

*Adjusted for age, sex, race, body mass index, marital status, education attainment, and poverty income ratio*.

**Table 4:** The associations of dioxins, furans, and coplanar PCBs metabolites with hypertension (*n* = 2,131).

	**OR**	**95%CI**	***P* value**
PCB66	1.21	1.02–1.43	0.027
PCB105	1.21	1.02–1.42	0.029
PCB128	1.08	1.00–1.16	0.037
3,4,4',5-tcb	1.24	1.02–1.51	0.036
1,2,3,6,7,8-hxcdd	1.17	1.03–1.33	0.015
1,2,3,4,6,7,8-hpcdd	1.36	1.07–1.72	0.013
1,2,3,4,6,7,8,9-ocdd	1.31	1.07–1.61	0.010
2,3,7,8-tcdd	1.49	1.19–1.87	0.001
1,2,3,7,8-pncdf	1.46	1.05–2.03	0.026
1,2,3,7,8,9-hxcdf	1.69	1.20–2.37	0.003
2,3,4,6,7,8-hxcdf	1.54	1.10–2.16	0.014
1,2,3,4,6,7,8,9-ocdf	1.35	1.12–1.63	0.003

*OR, odds ratio; CI, confidence intervals*.

*Adjusted for age, sex, race, body mass index, current smoking status, recreational physical activity, marital status, education attainment, and poverty income ratio*.

After excluding 7,997 hypertensive patients who were taking antihypertensive medication, we further examined the associations between household pesticide exposure and blood pressure levels. Overall, the systolic BPs in the pesticide-exposed and pesticide-unexposed groups were 120.8 ± 17.4 and 120.1 ± 16.9 mmHg, and the diastolic BPs were 70.7 ± 12.7 and 69.9 ± 12.6 mmHg, respectively. After adjusting for other covariates, household pesticide exposure was significantly associated with a high level of diastolic BP (β = 1.18, 95% CI: 0.64–1.73, *P* < 0.001), and marginally associated with systolic BP (β = 0.66, 95% CI: −0.01–1.33, *P* = 0.052) (Supplementary Table 4). Stratified analysis indicated a stronger association between household pesticide exposure and blood pressure level in current smokers than in non-smokers see (Supplementary Tables 5, 6), even if statistical significance was not reached (*P*_*interaction*_ = 0.750 for systolic BP and *P*_*interaction*_ = 0.088 for diabolic BP).

## Discussions

Our analysis shows that household pesticide exposure is significantly associated with hypertension. In addition, we report an interaction between smoking status and pesticide exposure on hypertension, indicating that smoking may accentuate the effect of pesticide exposure on BP.

In line with our results, previous studies reported that pesticide exposure was associated with hypertension (20–24). For example, Samsuddin et al. reported an association of chronic exposure to mosquito control-related pesticides with the increase in brachial and aortic BP in workers (25). Suarez-Lopez et al. found that pesticide exposure resulted in a short-term increase in both systolic and diastolic BP among children living in agricultural communities (26). It has also been shown that pregnant women who reported agricultural or domestic pesticide exposures had a higher risk of gestational hypertension than those unexposed (27, 28). Similar to our results, a systematic review and meta-analysis published in 2020, including 10 cross-sectional studies, six cohort studies, and one nested case-control study demonstrated that the population exposed to PCBs in the US were lower than those in other countries such as Japan and Italy, and PCBs metabolites exposure were positively associated with a high risk of hypertension (21). In a longitudinal study, Pavuk et al. also reported a positive association between dioxin and hypertension risk (7).

Previous studies reported that organophosphorus were positively associated with hypertension in humans (20, 26). Additionally, Bataillard et al. reported that low doses of organophosphorus, mainly including paraoxon and soman, provoked marked and durable hypertension in conscious unrestrained rats (29). Similar results were found in another experimental study on the effect of organophosphate chlorpyrifos (30). However, in this study, we found several negative associations for pyrethroids and organophosphate pesticides metabolites. Fish intake is a major source of exposure to lipophilic compounds; therefore, one possible explanation is that omega-3 fatty acids, a well-known protector for BP, reduce the negative effect of pesticides exposure (9, 31). In line with a previous study (32), we also reported phenolic compound was negatively associated with hypertension.

The mechanisms underlying the association between pesticide exposure and hypertension are still poorly understood. Adult mice perinatally exposed to dichloro-diphenyl-trichloroethane (DDT) exhibit chronically increased systolic BP, and elevated expression of mRNAs of several renal ion transporters. The increase in blood pressure was attenuated by treatment with an ACE inhibitor, suggesting that an over-activated renin/angiotensin system may be a key mechanism in DDT-induced hypertension (33). PCB126 induces changes characteristic for endothelial dysfunction in human essential hypertension through stimulating the production of vasoconstrictors such as COX-2, prostaglandins, and reactive oxygen species, and inhibiting the release of nitric oxide (34). *In vitro* experiment also showed that PCBs activates the poly (ADP ribose) polymerase, directly impairing endothelial cell functions, which may predispose exposed individuals to the development of hypertension (35). Our findings showed that PCBs were significantly associated with an elevated risk of hypertension.

The present study supports and extends previous findings by showing an interaction between smoking status and pesticide exposure on hypertension. This result is partially consistent with those obtained in previous studies. Lee et al. reported a difference between cigarette smoking status and total mortality, depending on serum concentrations of POPs among the elderly in the U.S. (14). This study found that among participants with relatively high POPs, former or current smokers had a higher risk of mortality than the never-smokers (14). The same research group further confirmed this interaction in another cohort of 70-year old participants in Sweden (13). However, the mechanisms responsible for this interaction effect have not been elucidated. One possible explanation is that pesticide components enter the body through smoking due to the pesticide residues from cigarettes. Indeed, exposure to pesticide residues in the adult population is twice as high for smokers than non-smokers (36). Another possible mechanism is that smoking cigarettes may compromise pesticide metabolism in the human body. In a study conducted among Norwegian women, smoking was significantly associated with higher levels of PCBs, p,p'-DDE, and β-HCH in breast milk (37). A cross-sectional study by Moon et al. including 401 participants found that participants who smoked fewer than 15 cigarettes/day had a higher risk of having high POP concentrations than never-smokers (38). Furthermore, experimental studies reported that pretreatment with some POPs increased the carcinogenicity of chemicals contained in cigarettes like benzopyrene, dimethylnitrosamine, and N-nitrosodiethlyamine (39, 40).

To the best of our knowledge, this is the largest study to estimate the association between household pesticide exposure and hypertension. Moreover, we report an interaction between smoking and household pesticide exposure on the risk of hypertension. The limitations of this study should also be noted. It is difficult to establish the cause-effect relationship using a cross-sectional design. On the other hand, cross-sectional analysis cannot examine the effect of long-term pesticide exposure on hypertension. Fewer hypertensive participants were smokers in this analysis, suggesting that persons are more likely to change their lifestyles once diagnosed with hypertension, which may bias the association. Another limitation is that the intensity and duration of exposure, the sensitivity of life period of exposure, different pesticide types, occupational exposure of pesticides, and distance to nearest farm data were not collected from the NHANES, and should be considered in future studies.

## Conclusion

In summary, our results suggest that household pesticide exposure is associated with an elevated risk of hypertension. We report that there is an interaction between smoking and household pesticide exposure on hypertension, suggesting that smoking might be able to accentuate the effect of pesticide exposure on BP. These findings indicated that household pesticides should be cautiously used due to their chronic effects on hypertension, especially for smokers. Further cohort studies are warranted to verify this association and elucidate the role of smoking as a potential effect modifier.

## Data Availability Statement

The datasets presented in this study can be found in online repositories. The names of the repository/repositories and accession number(s) can be found at: https://www.cdc.gov/nchs/nhanes/.

## Ethics Statement

The studies involving human participants were reviewed and approved by National Center for Health Statistics. The patients/participants provided their written informed consent to participate in this study.

## Author Contributions

HC performed the statistical analysis, interpreted the results, and wrote the first draft of the manuscript. XL, LC, LZ, and KC critically reviewed the manuscript. GH, YW, and SC contributed to the conceptualization of the analysis and interpretation of the results, and helped in writing the first draft of the manuscript and subsequent revisions. All authors contributed to the article and approved the submitted version.

## Conflict of Interest

The authors declare that the research was conducted in the absence of any commercial or financial relationships that could be construed as a potential conflict of interest.

## Publisher's Note

All claims expressed in this article are solely those of the authors and do not necessarily represent those of their affiliated organizations, or those of the publisher, the editors and the reviewers. Any product that may be evaluated in this article, or claim that may be made by its manufacturer, is not guaranteed or endorsed by the publisher.

## References

[1] Heffernan AL Baduel C Toms LM Calafat AM Ye X Hobson P. Use of pooled samples to assess human exposure to parabens, benzophenone-3 and triclosan in Queensland, Australia. Environ Int. (2015) 85:77–83. 10.1016/j.envint.2015.09.001 26368661 PMC5537729

[2] Larsson K Ljung Bjorklund K Palm B Wennberg M Kaj L Lindh CH. Exposure determinants of phthalates, parabens, bisphenol A and triclosan in Swedish mothers and their children. Environ Int. (2014) 73:323–33. 10.1016/j.envint.2014.08.014 25216151 PMC4207945

[3] Kim KH Kabir E Jahan SA. Exposure to pesticides and the associated human health effects. Sci Total Environ. (2017) 575:525–35. 10.1016/j.scitotenv.2016.09.009 27614863

[4] Freire C Koifman S. Pesticides, depression and suicide: a systematic review of the epidemiological evidence. Int J Hyg Environ Health. (2013) 216:445–60. 10.1016/j.ijheh.2012.12.003 23422404

[5] Pouchieu C Piel C Carles C Gruber A Helmer C Tual S. Pesticide use in agriculture and Parkinson's disease in the AGRICAN cohort study. Int J Epidemiol. (2018) 47:299–310. 10.1093/ije/dyx225 29136149

[6] Kalra S Dewan P Batra P Sharma T Tyagi V Banerjee BD. Organochlorine pesticide exposure in mothers and neural tube defects in offsprings. Reprod Toxicol. (2016) 66:56–60. 10.1016/j.reprotox.2016.09.005 27647593

[7] Pavuk M Serio TC Cusack C Cave M Rosenbaum PF Birnbaum LS. Hypertension in relation to dioxins and polychlorinated biphenyls from the anniston community health survey follow-up. Environ Health Perspect. (2019) 127:127007. 10.1289/EHP5272 31858832 PMC6957279

[8] Raffetti E Donato F De Palma G Leonardi L Sileo C Magoni M. Polychlorinated biphenyls (PCBs) and risk of hypertension: a population-based cohort study in a North Italian highly polluted area. Sci Total Environ. (2020) 714:136660. 10.1016/j.scitotenv.2020.136660 32018953

[9] Warembourg C Maitre L Tamayo-Uria I Fossati S Roumeliotaki T Aasvang GM. Early-life environmental exposures and blood pressure in children. J Am Coll Cardiol. (2019) 74:1317–28. 10.1016/j.jacc.2019.06.069 31488269 PMC8713646

[10] Goncharov A Pavuk M Foushee HR Carpenter DO Anniston Environmental Health Reseach Consortium. Blood pressure in relation to concentrations of PCB congeners and chlorinated pesticides. Environ Health Perspect. (2011) 119:319–25. 10.1289/ehp.1002830 21362590 PMC3059993

[11] Henriquez-Hernandez LA Luzardo OP Zumbado M Camacho M Serra-Majem L Alvarez-Leon EE. Blood pressure in relation to contamination by polychlorobiphenyls and organochlorine pesticides: Results from a population-based study in the Canary Islands (Spain). Environ Res. (2014) 135:48–54. 10.1016/j.envres.2014.05.036 25262074

[12] Agongo G Nonterah EA Amenga-Etego L Debpuur C Kaburise MB Ali SA. Blood pressure indices and associated risk factors in a rural West African adult population: insights from an AWI-Gen substudy in Ghana. Int J Hypertens. (2020) 2020:4549031. 10.1155/2020/4549031 32395338 PMC7201512

[13] Lee DH Lind L Jacobs DR Jr Salihovic S van Bavel B Lind PM. Does mortality risk of cigarette smoking depend on serum concentrations of persistent organic pollutants? Prospective investigation of the vasculature in Uppsala seniors (PIVUS) study. PLoS ONE. (2014) 9:e95937. 10.1371/journal.pone.0095937 24828407 PMC4020745

[14] Lee YM Bae SG Lee SH Jacobs DR Jr Lee DH. Associations between cigarette smoking and total mortality differ depending on serum concentrations of persistent organic pollutants among the elderly. J Korean Med Sci. (2013) 28:1122–8. 10.3346/jkms.2013.28.8.1122 23960436 PMC3744697

[15] National Center for Health Statistics. National Health and Nutrition Examination Survey. Available online at: https://www.cdc.gov/nchs/nhanes/index.htm (accessed May 1, 2019).

[16] Whelton PK Carey RM Aronow WS Casey DEJr. Collins KJ Dennison Himmelfarb C. 2017 ACC/AHA/AAPA/ABC/ACPM/AGS/APhA/ASH/ASPC/NMA/PCNA Guideline for the prevention, detection, evaluation, and management of high blood pressure in adults: executive summary: a report of the American College of Cardiology/American Heart Association Task Force on Clinical Practice Guidelines. Hypertension. (2018) 71:1269–324. 10.1161/HYP.0000000000000066 29133354

[17] Gabb GM Mangoni AA Arnolda L. Guideline for the diagnosis and management of hypertension in adults - 2016. Med J Aust. (2017) 206:141. 10.5694/mja16.01132 28208049

[18] Barr DB Olsson AO Wong LY Udunka S Baker SE Whitehead RD. Urinary concentrations of metabolites of pyrethroid insecticides in the general U.S. population: National Health and Nutrition Examination Survey 1999-2002. Environ Health Perspect. (2010) 118:742–8. 10.1289/ehp.0901275 20129874 PMC2898848

[19] Chen H Chen L Hao G. Exercise attenuates the association between household pesticide exposure and depressive symptoms: evidence from NHANES, 2005-2014. Environ Res. (2020) 188:109760. 10.1016/j.envres.2020.109760 32534257

[20] Hernandez-Mariano JA Baltazar-Reyes MC Salazar-Martinez E Cupul-Uicab LA. Exposure to the pesticide DDT and risk of diabetes and hypertension: Systematic review and meta-analysis of prospective studies. Int J Hyg Environ Health. (2022) 239:113865. 10.1016/j.ijheh.2021.113865 34700204

[21] Raffetti E Donat-Vargas C Mentasti S Chinotti A Donato F. Association between exposure to polychlorinated biphenyls and risk of hypertension: a systematic review and meta-analysis. Chemosphere. (2020) 255:126984. 10.1016/j.chemosphere.2020.126984 32679627

[22] Zago AM Faria NMX Favero JL Meucci RD Woskie S Fassa AG. Pesticide exposure and risk of cardiovascular disease: a systematic review. Glob Public Health. (2020) 8:1–23. 10.1080/17441692.2020.1808693 32816635

[23] Coletro HN Bressan J Diniz AP Hermsdorff HHM Pimenta AM Meireles AL. Total polyphenol intake, polyphenol subtypes, and prevalence of hypertension in the CUME cohort. J Am Coll Nutr. (2021). 10.1080/07315724.2021.1977735. [Epub ahead of print]. 34648393

[24] Miranda AM Steluti J Fisberg RM Marchioni DM. Association between polyphenol intake and hypertension in adults and older adults: a population-based study in Brazil. PLoS One. (2016) 11:e0165791. 10.1371/journal.pone.0165791 27792767 PMC5085083

[25] Samsuddin N Rampal KG Ismail NH Abdullah NZ Nasreen HE. Pesticides exposure and cardiovascular hemodynamic parameters among male workers involved in mosquito control in east coast of Malaysia. Am J Hypertens. (2016) 29:226–33. 10.1093/ajh/hpv093 26112865

[26] Suarez-Lopez JR Amchich F Murillo J Denenberg J. Blood pressure after a heightened pesticide spray period among children living in agricultural communities in Ecuador. Environ Res. (2019) 175:335–42. 10.1016/j.envres.2019.05.030 31150932 PMC6571166

[27] Ledda C Fiore M Santarelli L Bracci M Mascali G D'Agati MG. Gestational hypertension and organophosphorus pesticide exposure: a cross-sectional study. Biomed Res Int. (2015) 2015:280891. 10.1155/2015/280891 26339602 PMC4538315

[28] Murray J Eskenazi B Bornman R Gaspar FW Crause M Obida M. Exposure to DDT and hypertensive disorders of pregnancy among South African women from an indoor residual spraying region: the VHEMBE study. Environ Res. (2018) 162:49–54. 10.1016/j.envres.2017.12.006 29287179 PMC6118349

[29] Bataillard A Sannajust F Yoccoz D Blanchet G Sentenac-Roumanou H Sassard J. Cardiovascular consequences of organophosphorus poisoning and of antidotes in conscious unrestrained rats. Pharmacol Toxicol. (1990) 67:27–35. 10.1111/j.1600-0773.1990.tb00777.x 2395813

[30] Gordon CJ Padnos BK. Prolonged elevation in blood pressure in the unrestrained rat exposed to chlorpyrifos. Toxicology. (2000) 146:1–13. 10.1016/S0300-483X(00)00158-X 10773358

[31] Corcellas C Eljarrat E Barcelo D. First report of pyrethroid bioaccumulation in wild river fish: a case study in Iberian river basins (Spain). Environ Int. (2015) 75:110–6. 10.1016/j.envint.2014.11.007 25461420

[32] Parastar S Ebrahimpour K Hashemi M Maracy MR Ebrahimi A Poursafa P. Association of urinary concentrations of four chlorophenol pesticides with cardiometabolic risk factors and obesity in children and adolescents. Environ Sci Pollut Res Int. (2018) 25:4516–23. 10.1007/s11356-017-0771-y 29188597

[33] La Merrill MA Sethi S Benard L Moshier E Haraldsson B Buettner C. Perinatal DDT exposure induces hypertension and cardiac hypertrophy in adult mice. Environ Health Perspect. (2016) 124:1722–7. 10.1289/EHP164 27325568 PMC5089878

[34] Andersson H Garscha U Brittebo E. Effects of PCB126 and 17beta-oestradiol on endothelium-derived vasoactive factors in human endothelial cells. Toxicology. (2011) 285:46–56. 10.1016/j.tox.2011.04.003 21513769

[35] Helyar SG Patel B Headington K El Assal M Chatterjee PK Pacher P. PCB-induced endothelial cell dysfunction: role of poly(ADP-ribose) polymerase. Biochem Pharmacol. (2009) 78:959–65. 10.1016/j.bcp.2009.06.019 19549508 PMC2756480

[36] Lukyn MG Iwona H Mary GS Giulia V. Probabilistic modelling of exposure to pesticide residues in foods and tobacco. Int J Environ Agric Biotechnol. (2020) 5:261–74. 10.22161/ijeab.52.3

[37] Polder A Skaare JU Skjerve E Loken KB Eggesbo M. Levels of chlorinated pesticides and polychlorinated biphenyls in Norwegian breast milk (2002-2006), and factors that may predict the level of contamination. Sci Total Environ. (2009) 407:4584–90. 10.1016/j.scitotenv.2009.04.032 19457543

[38] Moon HJ Lim JE Jee SH. Association between serum concentrations of persistent organic pollutants and smoking in Koreans: a cross-sectional study. J Epidemiol. (2017) 27:63–8. 10.1016/j.je.2016.09.006 28142013 PMC5328728

[39] Diwan BA Ward JM Kurata Y Rice JM. Dissimilar frequency of hepatoblastomas and hepatic cystadenomas and adenocarcinomas arising in hepatocellular neoplasms of D2B6F1 mice initiated with N-nitrosodiethylamine and subsequently given Aroclor-1254, dichlorodiphenyltrichloroethane, or phenobarbital. Toxicol Pathol. (1994) 22:430–9. 10.1177/019262339402200409 7817132

[40] Hutton JJ Meier J Hackney C. Comparison of the *in vitro* mutagenicity and metabolism of dimethylnitrosamine and benzo[a]pyrene in tissues from inbred mice treated with phenobarbital, 3-methylcholanthrene or polychlorinated biphenyls. Mutat Res. (1979) 66:75–94. 10.1016/0165-1218(79)90010-7 106274

