# Supplementary material for: Associations Between Household Pesticide Exposure, Smoking and Hypertension
Source: Front Public Health. 2022 Feb 22;10:754643. doi: 10.3389/fpubh.2022.754643 (PMC8902065; doi:10.3389/fpubh.2022.754643)
Supplement: Supplementary file 1 [file Data_Sheet_1.docx]

Appendix A. Supplementary data

| eTable 1. The exposure levels of the metabolites for the different pesticides | | | |
| --- | --- | --- | --- |
| Variables | Normal | Hypertension | Total |
| **Chlorophenols (n=6651)** |  |  |  |
| 2,5-dichlorophenol(ug/L) | 9.45(8.8-10.14) | 10.43(9.67-11.25) | 9.91(9.41-10.44) |
| 2,4-dichlorophenol(ug/L) | 0.96(0.92-1.01) | 1.02(0.96-1.07) | 0.99(0.95-1.02) |
| **Carbamates (n=2686)** |  |  |  |
| Ethylene thiourea(ug/L) | 0.19(0.19-0.20) | 0.18(0.18-0.19) | 0.19(0.18-0.19) |
| Propylene thiourea(ug/L) | 0.26(0.25-0.26) | 0.25(0.25-0.25) | 0.25(0.25-0.26) |
| Sulfonylurea herbicides (n=2129) |  |  |  |
| Ethametsulfuron methyl(ug/L) | 0.07(0.07-0.07) | 0.07(0.07-0.07) | 0.07(0.07-0.07) |
| **Pyrethroids (n=7582)** |  |  |  |
| 3-phenoxybenzoic acid(ug/L) | 0.45(0.43-0.47) | 0.46(0.44-0.48) | 0.45(0.44-0.47) |
| **Organophosphate insecticides- diakyl phosphate metabolites (n=6782)** |  |  |  |
| Dimethylphosphate(ng/mL) | 1.31(1.24-1.38) | 1.38(1.30-1.45) | 1.34(1.29-1.39) |
| Diethylphosphate(ng/mL) | 0.78(0.74-0.82) | 0.85(0.80-0.90) | 0.81(0.78-0.85) |
| Dimethylthiophosphate(ng/mL) | 1.71(1.62-1.81) | 1.90(1.79-2.01) | 1.8(1.73-1.87) |
| Diethylthiophosphate(ng/mL) | 0.44(0.42-0.46) | 0.47(0.45-0.49) | 0.45(0.44-0.47) |
| Dimethyldithiophosphate(ng/mL) | 0.32(0.30-0.33) | 0.33(0.31-0.35) | 0.32(0.31-0.33) |
| Diethyldithiophosphate(ng/mL) | 0.14(0.13-0.14) | 0.14(0.13-0.14) | 0.14(0.14-0.14) |
| **Dioxins, furans, & coplanar PCBs (n=2131)** |  |  |  |
| PCB52 (ng/g) | 0.03(0.02-0.03) | 0.03(0.03-0.03) | 0.03(0.03-0.03) |
| PCB66 (ng/g) | 0.02(0.02-0.02) | 0.02(0.02-0.02) | 0.02(0.02-0.02) |
| PCB74 (ng/g) | 0.03(0.03-0.03) | 0.06(0.06-0.07) | 0.04(0.04-0.05) |
| PCB99 (ng/g) | 0.03(0.03-0.03) | 0.05(0.05-0.05) | 0.04(0.04-0.04) |
| PCB99 (ng/g) | 0.02(0.02-0.02) | 0.02(0.02-0.02) | 0.02(0.02-0.02) |
| PCB105 (ng/g | 0.01(0.01-0.01) | 0.02(0.02-0.02) | 0.02(0.02-0.02) |
| PCB118 (ng/g) | 0.04(0.04-0.04) | 0.08(0.07-0.08) | 0.05(0.05-0.06) |
| PCB128 (ng/g) | 0.00(0-0.01) | 0.01(0.01-0.01) | 0.01(0-0.01) |
| PCB138 (ng/g) | 0.10(0.09-0.10) | 0.19(0.18-0.20) | 0.14(0.13-0.14) |
| PCB146 (ng/g) | 0.02(0.02-0.02) | 0.03(0.03-0.04) | 0.03(0.02-0.03) |
| PCB153 (ng/g) | 0.13(0.12-0.14) | 0.28(0.26-0.29) | 0.19(0.18-0.20) |
| PCB156 (ng/g) | 0.02(0.02-0.02) | 0.04(0.04-0.04) | 0.03(0.03-0.03) |
| PCB157 (ng/g) | 0.01(0.01-0.01) | 0.02(0.01-0.02) | 0.01(0.01-0.01) |
| PCB167 (ng/g) | 0.01(0.01-0.01) | 0.02(0.01-0.02) | 0.01(0.01-0.01) |
| PCB170 (ng/g) | 0.04(0.04-0.04) | 0.08(0.08-0.09) | 0.06(0.06-0.06) |
| PCB172 (ng/g) | 0.01(0.01-0.01) | 0.02(0.02-0.02) | 0.01(0.01-0.01) |
| PCB177 (ng/g) | 0.01(0.01-0.01) | 0.02(0.02-0.02) | 0.02(0.02-0.02) |
| PCB178 (ng/g) | 0.01(0.01-0.01) | 0.02(0.02-0.02) | 0.02(0.01-0.02) |
| PCB180 (ng/g) | 0.09(0.08-0.10) | 0.21(0.20-0.22) | 0.14(0.13-0.14) |
| PCB183 (ng/g) | 0.01(0.01-0.02) | 0.03(0.02-0.03) | 0.02(0.02-0.02) |
| PCB187 (ng/g) | 0.03(0.03-0.03) | 0.06(0.06-0.07) | 0.04(0.04-0.05) |
| 1,2,3,7,8-pncdd (fg/g) | 16.67(15.81-17.57) | 27.42(25.96-28.95) | 21.43(20.6-22.29) |
| 1,2,3,6,7,8-hxcdd (fg/g) | 99.52(93.46-105.97) | 205.26(192.96-218.34) | 143.43(136.9-150.28) |
| 1,2,3,7,8,9-hxcdd (fg/g) | 24.28(23.48-25.1) | 33.28(31.94-34.67) | 28.47(27.7-29.26) |
| 1,2,3,4,6,7,8-hpcdd (fg/g) | 174.94(166.93-183.33) | 289.35(275.64-303.74) | 225.54(217.7-233.67) |
| 1,2,3,4,6,7,8,9-ocdd (fg/g) | 1361.5(1299.6-1426.35) | 2278.58(2169.28-2393.39) | 1765.81(1704.12-1829.73) |
| 2,3,7,8-tcdf (fg/g) | 11.05(10.81-11.3) | 11.79(11.53-12.06) | 11.42(11.24-11.6) |
| 1,2,3,7,8-pncdf (fg/g) | 11.81(11.54-12.08) | 12.62(12.34-12.91) | 12.21(12.02-12.41) |
| 2,3,4,7,8-pncdf (fg/g) | 24.28(23.23-25.37) | 39.65(37.86-41.53) | 31.1(30.07-32.16) |
| 1,2,3,4,7,8-hxcdf (fg/g) | 22.67(21.86-23.52) | 34.68(33.34-36.08) | 28.1(27.32-28.91) |
| 1,2,3,6,7,8-hxcdf (fg/g) | 19.68(19.02-20.36) | 28.49(27.4-29.61) | 23.72(23.09-24.37) |
| 1,2,3,7,8,9-hxcdf (fg/g) | 12.48(12.21-12.76) | 13.46(13.19-13.74) | 12.97(12.77-13.16) |
| 2,3,4,6,7,8-hxcdf (fg/g) | 12.86(12.56-13.17) | 14.35(14.02-14.69) | 13.59(13.37-13.82) |
| 1,2,3,4,6,7,8-hpcdf (fg/g) | 41.66(39.91-43.5) | 51.02(48.97-53.16) | 46.15(44.79-47.56) |
| 1,2,3,4,6,7,8,9-ocdf (fg/g) | 29.67(28.54-30.84) | 33.73(32.51-34.99) | 31.66(30.82-32.52) |
| 3,3',4,4',5-pncb (fg/g) | 97.37(92.44-102.56) | 175.94(165.72-186.79) | 131.27(125.92-136.84) |
| 3,4,4',5-tcb (fg/g) | 48.29(46.61-50.04) | 52.42(50.6-54.29) | 50.33(49.09-51.61) |
| 2,3,7,8-tcdd (fg/g) | 10.37(9.96-10.79) | 14.73(14.15-15.34) | 12.38(12.02-12.75) |
| 3,3',4,4',5,5'-hxcb (fg/g) | 64.93(61.57-68.47) | 124.92(118.92-131.21) | 90.35(86.92-93.92) |
| Geometric mean and its 95% confidence interval were reported. | | | |

| eTable 2. Associations between household pesticide exposure and hypertension using old guidelines | | | |
| --- | --- | --- | --- |
|  | OR | 95% CI | *P* value |
| **Model 1** |  |  |  |
| Control | Reference |  |  |
| Pesticide exposure | 1.01 | 0.93–1.11 | 0.749 |
| **Model 2** |  |  |  |
| Control | Reference |  |  |
| Pesticide exposure | 1.05 | 0.95–1.16 | 0.384 |
| **Model 3** |  |  |  |
| Control | Reference |  |  |
| Pesticide exposure | 1.06 | 0.96–1.17 | 0.235 |
| OR = Odds ratio; CI = confidence intervals  Model 1 unadjusted  Model 2 adjusted for age, sex, and race.  Model 3 adjusted for age, sex, race, body mass index, current smoking status, recreational physical activity, marital status, education attainment, and poverty income ratio.  Hypertension was defined as a mean systolic blood pressure of ≥140 mmHg, a mean diastolic blood pressure of ≥90 mmHg, or as “use of blood pressure -lowering medication”. | | | |

| eTable 3. The associations of different pesticide metabolites metabolites with hypertension | | | |
| --- | --- | --- | --- |
|  | OR | 95%CI | *P* value |
| **Chlorophenols(n=6651)** |  |  |  |
| 2,5-dichlorophenol | 0.95 | 0.92–0.98 | 0.002 |
| 2,4-dichlorophenol | 0.93 | 0.89–0.98 | 0.003 |
| **Carbamates (n=2686)** |  |  |  |
| Ethylene thiourea | 0.92 | 0.75–1.13 | 0.423 |
| Propylene thiourea | 1.44 | 0.02–105.05 | 0.864 |
| **Sulfonylurea herbicides (n=2129)** |  |  |  |
| Ethametsulfuron methyl | 1.53 | 0.40–5.80 | 0.520 |
| **Pyrethroids (n=7582)** |  |  |  |
| 3-phenoxybenzoic acid | 0.93 | 0.88–0.99 | 0.025 |
| **Organophosphate insecticides- diakyl phosphate metabolites (n=6782)** |  |  |  |
| Dimethylphosphate | 0.98 | 0.94–1.03 | 0.406 |
| Diethylphosphate | 0.99 | 0.95–1.04 | 0.808 |
| Dimethylthiophosphate | 1.01 | 0.97–1.05 | 0.662 |
| Diethylthiophosphate | 0.95 | 0.90–1.00 | 0.044 |
| Dimethyldithiophosphate | 0.97 | 0.92–1.03 | 0.310 |
| Diethyldithiophosphate | 0.99 | 0.90–1.09 | 0.814 |
| **Dioxins, furans, & coplanar PCBs (n=2131)** |  |  |  |
| PCB52 | 1.06 | 0.84–1.35 | 0.605 |
| PCB74 | 1.18 | 0.94–1.48 | 0.157 |
| PCB99 | 1.08 | 0.90–1.30 | 0.397 |
| PCB99 | 1.08 | 0.94–1.24 | 0.278 |
| PCB118 | 1.19 | 0.95–1.48 | 0.120 |
| PCB138 | 1.01 | 0.80–1.27 | 0.946 |
| PCB146 | 1.06 | 0.84–1.33 | 0.635 |
| PCB153 | 1.02 | 0.80–1.30 | 0.842 |
| PCB156 | 0.99 | 0.80–1.23 | 0.937 |
| PCB157 | 1.07 | 0.94–1.21 | 0.313 |
| PCB167 | 1.11 | 0.97–1.26 | 0.132 |
| PCB170 | 1.01 | 0.78–1.31 | 0.940 |
| PCB172 | 1.09 | 0.94–1.27 | 0.233 |
| PCB177 | 1.04 | 0.87–1.25 | 0.668 |
| PCB178 | 1.11 | 0.92–1.32 | 0.266 |
| PCB180 | 0.95 | 0.75–1.21 | 0.694 |
| PCB183 | 1.08 | 0.86–1.34 | 0.500 |
| PCB187 | 1.01 | 0.80–1.29 | 0.886 |
| 1,2,3,7,8-pncdd | 1.09 | 0.93–1.28 | 0.278 |
| 1,2,3,7,8,9-hxcdd | 1.27 | 0.96–1.68 | 0.092 |
| 2,3,7,8-tcdf | 1.45 | 0.99–2.13 | 0.056 |
| 2,3,4,7,8-pncdf | 1.17 | 0.98–1.39 | 0.082 |
| 1,2,3,4,7,8-hxcdf | 1.20 | 0.98–1.48 | 0.076 |
| 1,2,3,6,7,8-hxcdf | 1.26 | 1.00–1.60 | 0.052 |
| 1,2,3,4,6,7,8-hpcdf | 1.18 | 0.96–1.45 | 0.107 |
| 3,3',4,4',5-pncb | 1.16 | 0.97–1.38 | 0.106 |
| 3,3',4,4',5,5'-hxcb | 1.08 | 0.88–1.34 | 0.440 |
| OR = odds ratio, CI = confidence intervals.  Adjusted for age, sex, race, body mass index, current smoking status, recreational physical activity, marital status, education attainment, poverty income ratio, and creatinine in urine (except for dioxins, furans, & coplanar PCBs).  Log-transformations were applied for all the metabolites. | | | |

| eTable 4. The associations between household pesticide exposure and blood pressure | | | |
| --- | --- | --- | --- |
|  | β | 95%CI | *P* value |
| **Systolic blood pressure** |  |  |  |
| **Model 1** |  |  |  |
| Pesticide unexposed | Reference |  |  |
| Pesticide exposed | 0.56 | -0.22-1.34 | 0.158 |
| **Model 2** |  |  |  |
| Pesticide unexposed | Reference |  |  |
| Pesticide exposed | 0.73 | 0.02-1.44 | 0.043 |
| **Model 3** |  |  |  |
| Pesticide unexposed | Reference |  |  |
| Pesticide exposed | 0.66 | -0.01-1.33 | 0.052 |
| **Diastolic blood pressure** |  |  |  |
| **Model 1** |  |  |  |
| Pesticide unexposed | Reference |  |  |
| Pesticide exposed | 1.04 | 0.46-1.62 | 0.001 |
| **Model 2** |  |  |  |
| Pesticide unexposed | Reference |  |  |
| Pesticide exposed | 1.11 | 0.55-1.67 | <0.001 |
| **Model 3** |  |  |  |
| Pesticide unexposed | Reference |  |  |
| Pesticide exposed | 1.18 | 0.64-1.73 | <0.001 |
| CI=confidence intervals  Model 1 unadjusted  Model 2 adjusted for age, sex, and race.  Model 3 adjusted for age, sex, race, body mass index, current smoke, recreational physical activity, marital status, education attainment, and poverty income ratio. | | | |

| eTable 5. The associations between household pesticide exposure and systolic blood pressure by smoking status | | | |
| --- | --- | --- | --- |
|  | β | 95%CI | *P* value |
| **Smokers** |  |  |  |
| Pesticide unexposed | Reference |  |  |
| Pesticide exposed | 0.88 | -0.45-2.20 | 0.194 |
| **Non-smokers** |  |  |  |
| Pesticide unexposed | Reference |  |  |
| Pesticide exposed | 0.57 | -0.21-1.35 | 0.149 |
| CI=confidence intervals, RPA= recreational physical activity  Adjusted for age, sex, race, body mass index, marital status, education attainment, and poverty income ratio. | | | |

| eTable 6. The associations between household pesticide exposure and diabolic blood pressure by smoking status | | | |
| --- | --- | --- | --- |
|  | β | 95%CI | *P* value |
| **Smokers** |  |  |  |
| Pesticide unexposed | Reference |  |  |
| Pesticide exposed | 1.73 | 0.76-2.69 | 0.001 |
| **Non-smokers** |  |  |  |
| Pesticide unexposed | Reference |  |  |
| Pesticide exposed | 0.93 | 0.37-1.49 | 0.001 |
| CI=confidence intervals, RPA= recreational physical activity  Adjusted for age, sex, race, body mass index, marital status, education attainment, and poverty income ratio. | | | |
